# A Biomimetic Approach to Increasing Soft Actuator Performance by Friction Reduction

**DOI:** 10.3390/polym12051120

**Published:** 2020-05-14

**Authors:** Nguyen Quang Khuyen, Rudolf Kiefer, Fred Elhi, Gholamreza Anbarjafari, Jose G. Martinez, Tarmo Tamm

**Affiliations:** 1 Conducting Polymers in Composites and Applications Research Group, Faculty of Applied Sciences, Ton Duc Thang University, Ho Chi Minh City 700000, Vietnam; nguyenquangkhuyen@tdtu.edu.vn; 2 Intelligent Materials and System Lab, Institute of Technology, University of Tartu, Nooruse 1, 50411 Tartu, Estonia; elhi.fred@gmail.com (F.E.); tarmo.tamm@ut.ee (T.T.); 3 iCV Research Lab, Institute of Technology, University of Tartu, 50411 Tartu, Estonia; shb@ut.ee; 4 Faculty of Engineering, Hasan Kalyoncu University, 27100 Gaziantep, Turkey; 5 Division of Sensor and Actuator Systems, Department of Physics, Chemistry and Biology (IFM), Linköping University, 581 83 Linköping, Sweden; jose.gabriel.martinez.gil@liu.se

**Keywords:** PDMS, PET-PPy, bilayer, actuation efficiency, contact angle, hydrophobic surface, reduction of friction

## Abstract

While increasing power output is the most straight-forward solution for faster and stronger motion in technology, sports, or elsewhere, efficiency is what separates the best from the rest. In nature, where the possibilities of power increase are limited, efficiency of motion is particularly important; the same principle can be applied to the emerging biomimetic and bio-interacting technologies. In this work, by applying hints from nature, we consider possible approaches of increasing the efficiency of motion through liquid medium of bilayer ionic electroactive polymer actuations, focusing on the reduction of friction by means of surface tension and hydrophobicity. Conducting polyethylene terephthalate (PET) bilayers were chosen as the model actuator system. The actuation medium consisted of aqueous solutions containing tetramethylammonium chloride and sodium dodecylbenzenesulfonate in different ratios. The roles of ion concentrations and the surface tension are discussed. Hydrophobicity of the PET support layer was further tuned by adding a spin-coated silicone layer to it. As expected, both approaches increased the displacement—the best results having been obtained by combining both, nearly doubling the bending displacement. The simple approaches for greatly increasing actuation motion efficiency can be used in any actuator system operating in a liquid medium.

## 1. Introduction

Soft, compliant ionic electroactive polymer actuators are expected to find applications in many fields, from micro machining [1] for valves and pumps [2,3], simultaneous sensors and actuators [4], lab on chip devices [5], smart textiles [6], and soft robotics [7] to biomedical devices [8]. Typically, higher displacements, more force, and faster actuation have been sought; however, increasing just the power output can be tricky, as increased charge consumption drains batteries, brings along unnecessary heating, and can also lead to material degradation.

Alternatively, performance increase can be obtained by increasing efficiency, conserving precious energy. Reduction of friction is a common approach in many fields of engineering and technology. Using hydrophobic polymeric surfaces such as those of PDMS [9,10] is one of the mayor approaches to increasing the motion of fluids in micro fluidics [11] for micro-electro-mechanical system applications [12], biomedical applications [13], electrowetting applications [14], and switchable adhesion surfaces in robotic devices leaning on the gecko-feet adaptation [15].

Nature has introduced a large variety of techniques for ensuring optimal surface interactions; hydrophobic (often superhydrophobic, applying hierarchical microstructure and air retention) surfaces are made use of by representatives of both fauna and flora [16]; the Nepenthes pitcher plants are a well-known example of applying lubrication to already slippery surfaces to effectively catch insects [17]. An increasing number of bio-mimetic surfaces have been developed in recent years in attempt to obtain non-wetting or non-fouling surfaces or reduce friction [18]. It is perhaps somewhat surprising that the growing understanding of friction, in particular, that of water-based and charged systems [19], has not been applied in the context of soft actuator motion in electrolyte solutions; as with miniaturization, the efficiency and surface interactions become critical.

In the following, we describe approaches to increasing the efficiency of soft ionic electroactive polymer actuators in aqueous electrolyte solutions using a simple polyethylene terephthalate (PET) –polypyrrole (PPy) bending double layer as the model system. The electroactive PPy layer doped with dodecylbenzenesulfonate (PPy/DBS) was deposited electrochemically on a conductive coating of poly (3,4-ethylenedioxythiophene) poly(styrenesulfonate)/multiwall carbon nanotubes (PEDOT:PSS/MWCNT) painted on a PET sheet, as described before [20]. In the case of PPy/DBS, the actuation is a result of volume change due to the flux of cations during the redox switching to maintain electroneutrality as the DBS^−^ anions are immobilized in the PPy network [21,22,23]. The charge efficiency of the system is dependent on the interaction of the polymer matrix and the cations, the size of the solvated ions [24] making an important contribution. Other factors to consider for increased displacement are the polymerization conditions [25,26], the electrolyte choice [27], the carbon-based subsidies [28], and the addition of ion conductive additives such as polyethylene oxide [29] or the use of ionic liquids [30]. Naturally, the ratio of the working material thickness to that of the passive substrate should be sufficient [31]. There have, however, been a few attempts to modify the performances of bending piezoelectric actuators by special back-side coatings [32,33].

As a PET-PPy/DBS bilayer bends in an aqueous electrolyte, it has to apply force against the water resistance, consuming energy, which results in suppressed displacement of the bilayer. Our goal in this work was to see if it is possible to minimize the liquid media resistance to bending of the actuator by increasing the actuator hydrophobicity via applying a backside coating of polydimethylsiloxane (PDMS). Hydrophobic PDMS surfaces have been applied in microchannels [34] to increase the flow in microfluidics [35] devices, as the Milli-Q water-PDMS interface has a rather low contact angle, in the range of 100–110 degrees [36].

## 2. Experimental

### 2.1. Materials

Sodium dodecylbenzenesulfonate (NaDBS, 99%), multiwalled carbon nanotubes (MWCNT, 95%), polydimethylsiloxane (SYLGARD^®^ 184, 6500 cSt), and pyrrole (98%) in analytical grade from Sigma-Aldrich (Taufkirchen, Germany) were applied without further purifications. Tetramethylammonium chloride (TMACl, 98%) was from Fluka (Darmstadt, Germany); poly-3,4-ethylenedioxythiophene poly(styrenesulfonate) (PEDOT-PSS) at a concentration of 1.2–1.4% (in H_2_O) was from AGFA (S-200-G3, Mortsel, Belgium); and polyethylene terephthalate (PET, thickness 9 µm) was from Arsh Tradex Ltd. (Delhi, India). Milli-Q water (high-resistance ultra-purified water) was used as supplied.

### 2.2. Conductive and Hydrophobic Coatings

One gram of the main component and 0.1 g of curing agent of SYLGARD^®^ 184 were dissolved in hexane (48.9 g) and ultra-sonicated for 20 min as described elsewhere [37]. The solution was then spin coated at 5000 rpm (WS-650-23 spin coater, Laurell technology, North Wales, PA, USA) for 2 min to obtain a uniform film of 0.5 µm thickness on one side of a PET-sheet; then cured in the oven at 100 °C for 2 h. To obtain a stable suspension of MWCNT, the material in 1 wt% concentration was ultra-sonicated (Sonifier 250, Branson Ultrasonics Corporation, Danbury, Connecticut, USA) for 1 h, (50 Hz) in 0.1 M NaDBS. PEDOT-PSS:MWCNT (5.9:1) was spin coated (5000 rpm, 2 min) to obtain 0.5 µm thick films on the other side of PET-sheets with and without PDMS coating on the other side. The conductively coated PET was dried in the oven at 100 °C for 1 h.

### 2.3. Polymerization of PPy; Actuation of PET-PPy Bilayer

The PET sheets (length: 3 cm, width: 1 cm) with conductive coatings were placed as working electrodes together with a stainless steel counter electrode and a Ag/AgCl (3M KCl) reference electrode in 0.1 M Py and 0.1 M NaDBS_aq_. Under galvanostatic conditions (0.2 mA cm^−2^ for 2000 s, at 25 °C) PPy was deposited, yielding PET-PPy bilayers with and without PDMS backside coatings. The actuation solution contained various concentrations of TMACl and DBS in a three-electrode set-up (Figure S1). To ensure that no water migrated to the contact of the working electrode during the measurements, an enamel transversal paint strip was applied between the electrical clamp and the electrolyte meniscus in order to avoid direct contact between the aqueous electrolyte and the clamp [38]. The actuation was recorded as a video with a CCD camera (Sony Cyber-shot DSC-F717, Tokyo, Japan); the angular motion [39,40] of the bilayers in time was obtained by analyzing individual frames using Matlab-based in-house software [41,42].

### 2.4. Electrochemical Measurement Techniques

The electro-chemo-mechanical responses of the bilayer samples were investigated by applying electrochemical techniques (Eco Chemie Autolab PGSTAT30 potentiostat/galvanostat, Utrecht, Netherlands) of cyclic voltammetry (scan rate 10 mV/s, ±0.85 V) and potential steps (frequency range 0.017–1 Hz, ±0.85 V). From each bilayer type, at least three samples were measured independently; the actuation results are presented as mean values with standard deviations.

### 2.5. Characterization

The angular displacements of the bilayers were measured in at least triplicates in the potential range of ±0.85 V in mixed electrolyte systems (aqueous TMACl + NaDBS). Cyclic voltammetry and square wave potential steps were applied to investigate the actuation properties of PET-PPy and (PDMS) PET-PPy bilayers. The hydrophobicity of the PDMS layer was characterized by contact angle measurements; surface tension measurements were performed to investigate the electrolyte mixtures. Scanning electron microscopy images and conductivity measurements were carried out to characterize the deposited PPy/DBS on the flexible PET and (PDMS) PET sheets.

The surface tension of the aqueous electrolytes was measured at 25 °C and atmospheric pressure using Wilhelmy plate apparatus (Tensiometer Model K12, Krüss, Hamburg, Germany). Contact angle measurements (DSA-30, Krüss, Hamburg, Germany) of Milli-Q water and the electrolyte solutions were made on coated and uncoated PET-surfaces, and pure PDMS layers were obtained from mold to investigate the hydrophobicity. To measure conductivity, a four-point conductivity meter was applied (Model RM2, Jandel 4-Point Probe Head, Leighton Buzzard, UK). The images of the surfaces and cross-sections (samples broken under liquid nitrogen to keep the cross-sections as unmodified as possible) of the different conductive and hydrophobic coatings and the polymerized bilayer were characterized using scanning electron microscopy (SEM) (Helios NanoLab 600, Hillsboro, OR, USA).

## 3. Results and Discussion

A large number of publications have presented attempts to achieve higher performance from soft bending actuators by altering the composition of the actuators themselves. On the contrary, our experiments with bilayers of PPy/DBS deposited on conductive PET sheets were carried out in order to attempt efficiency improvement by reduction of friction. To that end, we have varied the solution blend composition of TMACl and NaDBS, where NaDBS acts as a surfactant reducing the surface tension of the electrolyte solution. Additionally, hydrophobic PDMS coatings on backside of the PET-PPy bilayer were applied to further reduce the friction in the electrolyte solution.

### 3.1. PPy/DBS Electropolymerization

**Figure 1:**
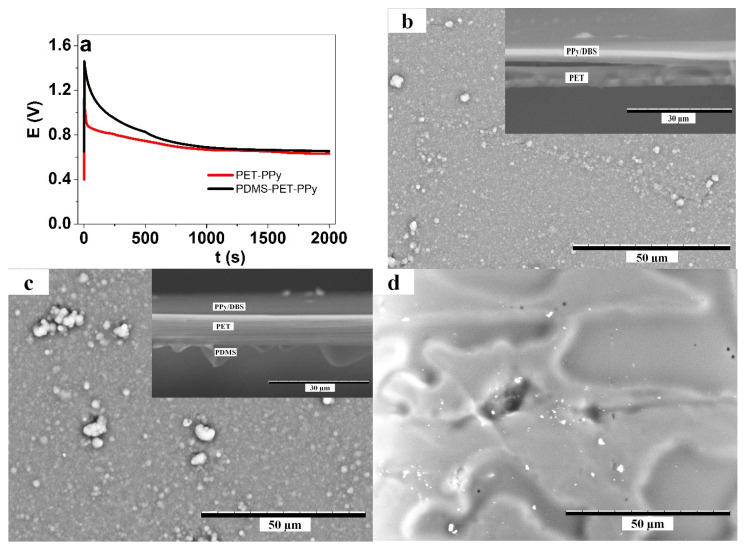
PPy deposited on PET and (PDMS) PET sheets with conductive coatings based on PEDOT: PSS/MWCNT showing (**a**) the galvanostatic polymerization (0.2 mA cm^−2^) at room temperature obtaining PET-PPy (red line) and (PDMS) PET-PPy (black line) bilayers. The SEM surface images (scale bar 50 µm) presenting (**b**) the surface morphologies of PET-PPy (inset cross section, scale bar 30 µm) and (**c**) (PDMS) PET-PPy (surface and cross section). (**d**) The surface image of PDMS coatings on PET.

To ensure reproducibility of the results, all materials were fabricated and tested in at least triplicates. The galvanostatic polymerization response of PPy doped with DBS on conductive (PEDOT: PSS/MWCNT) PET and (PDMS) PET is shown in Figure 1a. The resulting SEM images of surface and cross sections are shown in Figure 1b–d.

The polymerization curve (Figure 1a) shows that in both cases, after an initial peak, the potential decreases (faster in the case of the bilayer without PDMS, pointing to an easier/lower energy polymerization for PET-PPy) to form a plateau of about 0.65 V for both cases. The SEM images in Figure 1b,c show smooth surface morphology of PPy. The insets in Figure 1b,c show the cross section of the bilayer, revealing a PPy layer and a PDMS layer. The two different bilayer types demonstrate that the deposits of PPy are quite similar in surface morphology. Figure 1d shows the surface of the thin coated PDMS bilayer on PET. The electronic conductivity of deposited PPy/DBS on both bilayer types was found in similar range of 31 ± 3 S cm^−1^ as reported before [20]. Therefore, it could be concluded that the backside treatment with PDMS had virtually no effect on PPy deposition or properties, as expected.

### 3.2. Friction Reduction

#### 3.2.1. Electrolyte Concentration and Electrolyte Blends

The surface tension reducing effect or the surfactant NaDBS in concentration of 0.05 M was studied for different concentrations of TMACl (0.05, 0.1, 0.2, and 0.5 M) aqueous solutions. The surface tension values of the electrolytes are presented in Table 1.

The surface tension of Milli-Q water was found to be in range of 70.1 mN m^−1^, close to a literature value of 72 mN m^−1^ [43]. With TMACl (0.05 M), the surface tension of the aqueous solution increased slightly to 72.2 mN m^−1^. Adding NaDBS (0.05 M) in Milli-Q reduced the surface tension considerably to 30.9 mN m^−1^. In combined solutions with constant NaDBS concentration (0.05 M), increased TMACl concentrations led to very slight decrease of surface tension. Lower surface tension seen from mixed electrolyte of the electrolyte we assume will reduce the pushing force of a bilayer in actuation due to reduction of friction.

#### 3.2.2. PDMS Coatings on PET Layer

To reduce the friction of the PET layer in the aqueous solutions, a thin layer of PDMS (~0.5 µm) on the backside of PET-PPy bilayers was employed. While hierarchically structured surfaces with air trapping could in principle provide a greater effect, we have used the simplest possible approach to demonstrate the concept, and also for increased reproducibility.

#### 3.2.3. Contact Angle for Combined Approach

A logical step further was to combine both friction reduction approaches: surfactant and hydrophobic coating. The measured contact angles of pure water and electrolyte solutions on PET and PDMS are shown in Table 2.

As seen from Table 2, the contact angles of both water (and TMACl in water) and the mixed electrolyte solution were clearly lower on PET than on PDMS, with nearly equal shift of 53°. The water-on-PDMS result of 113° agrees well with literature values [36] in range of 110°. For water, the contact angle further increased on PDMS coated on PET to reach 117.6°, which was attributed to rougher morphology (Figure 1d) of the coating compared to cast film. For the mixed electrolyte, the contact angle on PET-PDMS was significantly lower than on freestanding PDMS, but still much higher than on pure PET. The reduced effect of PDMS on PET was attributed to the amphiphilic nature [44] of the mixed electrolyte, with the NaDBS component as a detergent [45]. 

### 3.3. Effect of Reduced Friction on Actuation

#### 3.3.1. Effect of Electrolyte Composition

As seen above, the presence of NaDBS in the electrolyte solution reduced the surface tension considerably. To see whether the lowered surface tension has an effect on the actuation response, and if so, how much, first, the response to square wave potential steps in frequency range 0.017–1 Hz was investigated.

**Figure 2:**
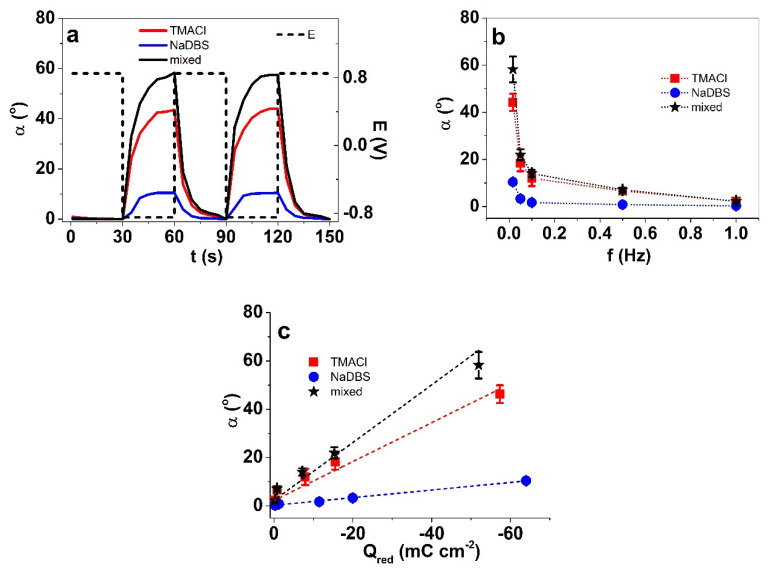
Square wave potential step measurements of PET-PPy bilayer in potential range ±0.85 V showing in (**a**), the displacement angle α and potential E (dashed) against time t of two subsequent cycles (fourth–fifth) at 0.017 Hz in aqueous electrolytes containing 0.05 M TMACl (red line), 0.05 M NaDBS (blue line), and mixed 0.05 M TMACl + 0.05 M NaDBS (black line). The dependencies of displacement on frequency of the PET-PPy bilayers in TMACl (■), NaDBS (●), and mixed (★) are shown in (**b**), and the displacements of the samples against the charge densities upon reduction (Q_red_) are presented in (**c**). The dashed curves in (**c**) represent the linear fits, shown here for orientation.

Solutions of TMACl, NaDBS, and the mixed TMACl + NaDBS were compared. The displacement α (°) against time t of two subsequent cycles at the applied frequency 0.017 Hz for PET-PPy bilayers in different electrolyte solutions are shown in Figure 2a; the dependency of displacement on the applied frequency is shown in Figure 2b; and the relation of charge density at reduction to displacement is presented in Figure 2c. 

The peak PET-PPy bilayer displacements (Figure 2a) for bilayer actuators moving in 0.05 M TMACl, 0.05 M NaDBS, and mixed (TMACl + NaDBS) solutions were 44°, 10°, and 58.2°, respectively. The PPy/DBS films are cation-driven actuators following Equation (1) [46,47]. The embedded-during-synthesis DBS^−^ anions are immobile, forcing solvated cations C^+^ (TMA^+^) to be incorporated during reduction to balance the excess negative charge.

(1) $$\begin{array}{r} {}[\left({\mathrm{PPy}}^{n+}\right)\left({\mathrm{DBS}}^{-}{)}_{n}\right]+n\left(C^{+}\right)+m\left(S\right)+n\left(e^{-}\right)\\ ⇌[\mathrm{PPy}{)}^{0}{\left({\mathrm{DBS}}^{-}\right)}_{n}\left(C^{+}\right)\left(S{)}_{m}\right] \end{array}$$

It is well known that DBS^−^ anions are amphiphilic anions, and while entrapped in PPy network (Equation (1)) have their polar end (the sulfonate) towards the oxidized PPy and a hydrophobic chain compatible with the neutral (reduced) PPy [21]. To balance the residual negative charge of DBS^−^ anions upon reduction (right end of Equation (1)), cations C^+^, TMA^+^ (in TMACl) or Na^+^ (NaDBS) enter the PPy film. It is important to mention that TMA^+^ represents an apolar ion with a more hydrophobic nature [48] and a charge sterically hidden away, leading to barely any solvation shell in aqueous solutions compared to the strongly-hydrated alkali cations (such as Na^+^). Consequently, the TMA^+^ cations enter the PPy/DBS polymer film faster/more easily, as seen from the much faster onset of bending angle in case of TMACl-containing solutions compared to pure NaDBS (Figure 2a); during the opposite half-cycle, there is no such difference (exit is fast for both cations). The faster response is also obvious from the frequency responses (Figure 2b); the response of NaDBS remains flat and minimal above 0.1 Hz, whereas the response in TMACl-containing solutions increases with lowered frequency throughout. Not just being faster, the TMA^+^ cations of size 0.274 nm [49] are also virtually without a solvation shell; therefore, they can interact more favorably with the hydrophobic tails of the DBS^−^ dopant anions and the reduced PPy, leading to about four times higher bending angle of PET-PPy bilayer in aqueous TMACl than in NaDBS.

Figure 2c presents the angular displacement against the charge density at reduction for all three different electrolytes. Conducting polymers are faradaic actuators [50]; the charge density determines the actuation displacement for each particular system; therefore, the linear relation between charge density and displacement with higher displacement at lower frequency (Figure 2b) is expected. It may seem a bit surprising that while the displacement was lowest in NaDBS, the obtained charge densities in that electrolyte were the highest. This apparent discrepancy can be explained by the break-down of solvation shells of Na^+^ cations upon entering the polymer film. As seen also from the bending angle response, the initial entrance of the Na^+^ cations was delayed, but once the driving force gets large enough, they start to enter, likely leaving their solvation shell behind (the exit is fast). Once without, they are small and mobile enough to allow high charge densities to be achieved, though without much mechanical coupling, as the accompanied displacement is low. The lowest charge densities of the three, especially clearly observed at the lowest frequency (0.017 Hz) corresponding to the highest current densities, were those of the mixed electrolyte. With a higher (total) ion concentration, the opposite could have been expected. Apparently, the hydrophilic Na^+^ and hydrophobic TMA^+^ cations interfere with each-other’s flux. As increased charge density was ruled out as the cause of increased bending angle in the mixed electrolyte, the possible explanation is the reduction of the surface tension—the resistance to displacement. To see if the ratio of TMACl:NaDBS played a role in the charge density and bending response, the concentration of TMACl in the mixture was varied (Table 3).

At both shown frequencies, the lowest TMACl concentration (highest NaDBS:TMACl ratio) yields in highest displacement (Table 3). The charge density peaks at 0.1 M TMACl concentration lead to a charge efficiency drop. For a different PPy/DBS system and different electrolyte (NaCl), optimal electrolyte concentration was also observed recently [27]. The occurrence of the optimum was explained by the lack of “free” water in solution at higher concentrations, leading to decreased osmotic pressure [27]. Both experimental and theoretical works [51] have shown that in case of multiple species of hydrophilic and hydrophobic ions, the clustering becomes rather complex, and changes the electric double layer structure and thickness.

Overall, the results showed that while the main expansion at reduction in the mixed electrolyte comes from TMACl, the addition of NaDBS leads to lowered charge density but increased bending displacement, potentially explained by lowered surface tension.

#### 3.3.2. Hydrophobic PDMS Coatings

**Figure 3:**
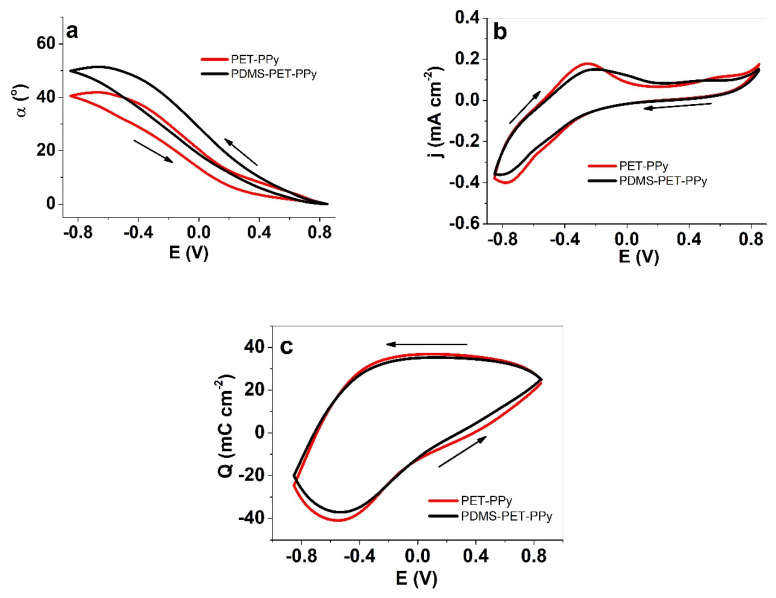
Cyclic voltammetry (10 mV s^−1^, third cycle) of PET-PPy (red) and (PDMS)PET-PPy (black) bilayers in aqueous 0.05 M TMACl showing (**a**) the angular displacement *α*, (**b**)the current density *j*, and (**c**) the charge density *Q* against the potential *E* (±0.85 V) against Ag/AgCl (3M KCl) reference electrode. The arrows indicate the start and ending of the cycle.

The response of the (PDMS) PET-PPy in comparison of PET-PPy to cyclic voltammetry in TMACl electrolyte solution is shown in Figure 3.

While there were no significant qualitative changes in the voltammograms for current or charge density response (Figure 3b), the actuation (angular displacement) of (PDMS) PET-PPy reached 50°, an increase of about 25% over PET-PPy (Figure 3a). As the charge density was even slightly lower in case of (PDMS) PET-PPy (potentially due to the working layer moving further away from the counter electrode), the charge efficiency was increased (Figure 3c). With other parameters virtually unchanged, the increase of bending angle can only be explained by the reduced friction of the actuator while moving through the solvent caused by the PDMS layer.

**Figure 4:**
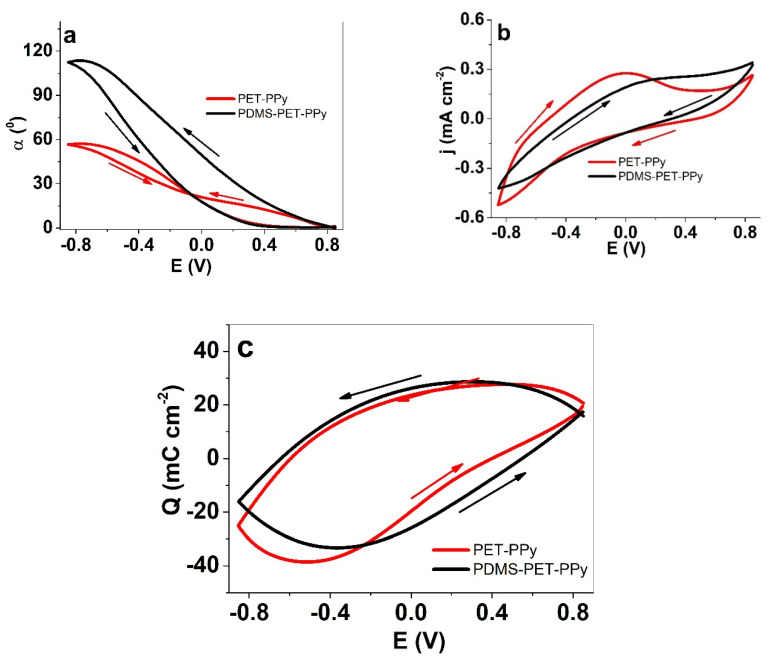
Cyclic voltammetry (scan rate 10 mV s^−1^, third cycle) in potential window of ±0.85 V in aqueous mixed electrolyte (TMACl + NaDBS) of PET-PPy (red line) and (PDMS) PET-PPy bilayer (black line) showing (**a**) the angular displacement *α*, (**b**) the current density *j*, and (**c**) the charge density *Q* against potential *E*. The arrows indicate the direction of the scan.

**Figure 5:**
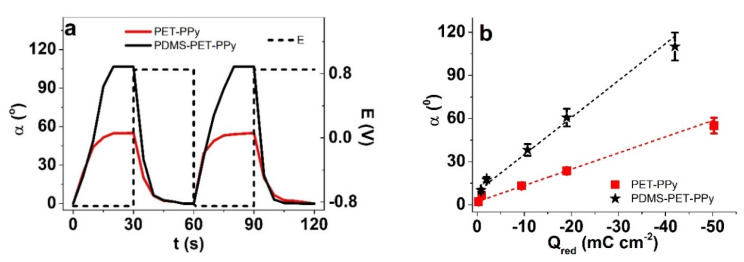
Square wave potential step measurements (aqueous NaDBS + TMACl, ±0.85 V) with angular displacement *α* of bilayers showing (**a**) two subsequent cycles (third-fourth cycle, 0.017 Hz) of PET-PPy bilayer (red line) and (PDMS)PET-PPy (black line) with applied potential *E* against time *t*. The angular movement *α* of PET-PPy (■) and (PDMS) PET-PPy (★) against charge density *Q_red_* upon reduction is shown in (**b**).

In the mixed electrolyte, all of the above-seen effects became even more pronounced, as seen from the cyclic voltammetry (Figure 4) and square wave potential steps’ (Figure 5) results. The images of the bilayer motion are shown in Figure S2.

By combining both the backside surface treatment by PDMS coating and the mixed electrolyte approach, the displacement was greatly increased, reaching 112°—a 1.9-times improvement over uncoated PET-PPy in the same solution (Figure 4a). The lowering of the current (Figure 4b) and charge densities (Figure 4c) was slightly more pronounced here for the PDMS-coated bilayers than above, while both the current density and charge density response had reduced (Faradaic) features in their shapes. Clearly, the effect of the hydrophobic PDMS coating is much larger in the mixed electrolyte solution. This superposition of the effects can be explained by the interaction of the amphiphilic electrolyte with the hydrophobic PDMS coating, emerging in a highly slippery interface [13], similarly to lubrication effects of the Nepenthes pitcher plants, which apply the infusion of a lubricating hydrophobic liquid into a hydrophobic rough substrate to achieve super-slippery surfaces [52]. Interestingly, in the present case, similar phenomena appear to take place in a solution.

The increased displacement at reduced charge densities is perhaps even more clearly observed from square wave potential steps experiments (Figure 5). The saturation of displacement was reached for both coated and uncoated bilayers in just 20 s, indicating a relatively high mobility of the cations in the PPy/DBS and no high entrance barrier (Figure 5a).

The PDMS coating on the PET-PPy bilayer increased the angular displacement from 55° to 108°, a similar increase to the one observed in cyclic voltammetry. Interestingly, the increase of displacement (1.9 times) shown in Figure 5b (Figure S3 revealed the angle against frequency) is numerically, again, about the same as the contact angle difference between PET and PDMS on PET (Table 2).

## 4. Conclusions

In the present work, two approaches were analyzed for increasing the actuation response of bilayers bending in electrolyte solutions. The aim was to achieve an improved response not by increasing power (current density, voltage) but with increased efficiency of motion by decreasing friction. The inclusion of a surface tension lowering agent (NaDBS) in the aqueous TMACl electrolyte solution achieved a clear increase in angular displacement by 33%. A much more significant increase of up to 1.9 times was achieved in the mixed solution by a simple layer of hydrophobic PDMS coated on the backside of the bilayer samples. Interestingly, in just TMACl solutions, the displacement increase due to the PDMS coating was just 20%. The combined hydrophobic surface + added surfactant approach can be observed in nature as well; for instance, on Nepenthes pitcher plants. The reduction of friction of soft actuators in aqueous electrolytes should be considered for any potential applications, as the increase in actuation was not accompanied by increased charge consumption, thereby greatly increasing (charge) efficiency. These results will give a new direction to improving the responses of soft bending actuators in various soft robotics and micro-actuator applications.

## Figures and Tables

**Table 1:** Surface tension of aqueous electrolytes.

Medium	Surface Tension, γ [mN m^−1^]
H_2_O (Milli-Q)	70.1 ± 2.3
TMACl (0.05 M)	72.2 ± 5.3
NaDBS (0.05 M)	30.9 ± 2.2
NaDBS (0.05 M)	
+0.05 M TMACl	31.3 ± 1.3
+0.1 M TMACl	30.5 ± 2.4
+0.2 M TMACl	30.3 ± 1.9
+0.5 M TMACl	29.9 ± 1.1

**Table 2:** The contact angles of Milli-Q water and a mixed electrolyte solution (0.05 M TMACl + 0.05 M NaDBS) on various surfaces. TMACl solutions without NaDBS had very similar values to pure Milli-Q.

Surface	Milli-Q [°]	Electrolyte Blend [°]
PET	59.7 ± 3.3	19 ± 1.2
PDMS	113 ± 7.2 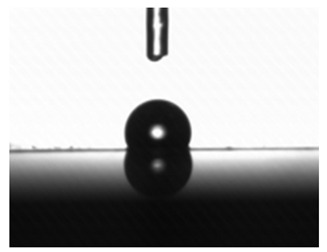	73 ± 5.4 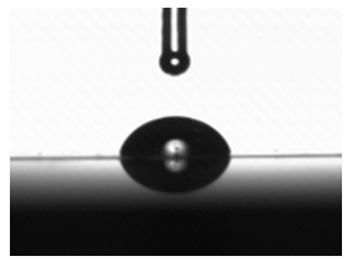
PET-PDMS	117.6 ± 8.6 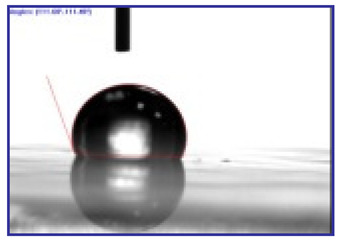	51.8 ± 3.3 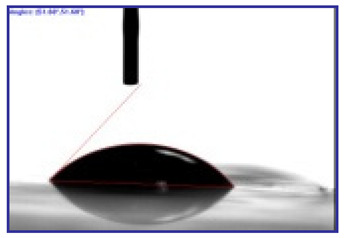

**Table 3:** PET-PPy bilayer displacement and charge density in mixed electrolytes with constant NaDBS and varied TMACl concentration.

Mixed Electrolyte NaDBS +	Displacement α [°]		Charge Density Q_red_ [mC cm^−2^]	
	0.017 Hz	0.1 Hz	0.017 Hz	0.1 Hz
0.05 M TMACl	58.2 ± 5.5	14.1 ± 1.2	−51.9 ± 4.9	−7.2 ± 0.6
0.1 M TMACl	45.6 ± 4.3	8.3 ± 0.7	−59.9 ± 5.4	−8.1 ± 0.8
0.2 M TMACl	36.3 ± 3.3	7.7 ± 0.9	−56.0 ± 5.2	−7.4 ± 7.1
0.5 M TMACl	24.2 ± 2.5	4.1 ± 0.5	−48.5 ± 4.3	−5.5 ± 0.6

## Supplementary Materials

The following are available online at https://www.mdpi.com/2073-4360/12/5/1120/s1, Figure S1. Set up of the bending beam measurements applying the bilayer samples as working electrode with counter electrode a platinum sheet and an Ag/AgCl (3M KCl) reference electrode in aqueous electrolyte, Figure S2. Videoframes of angular displacement under cyclic voltammetric measurements (scan rate 10 mV s^−1^) in binary aqueous electrolyte (0.05 M NaDBS + 0.05 M TMACl) with Pt counter electrode (CE) and Ag/AgCl (3M KCl) reference electrode (RE) showing the images of a: PET-PPy bilayer at −0.85 V, b: PET-PPy-bilayer at +0.85 V, c: (PDMS)PET-PPy bilayer at −0.85 V and PDMS-PET-PPy bilayer at +0.85 V. The position of the PET side and PPy/DBS side is shown in (a) and for (PDMS)PET-PPy bilayer in (c), Figure S3. The angular displacement α of PET-PPy (■) and PDMS-PET-PPy (★) at square wave potential steps against the applied frequency f (0.017 Hz to 1 Hz).
