# Supplementary material for: A Biomimetic Approach to Increasing Soft Actuator Performance by Friction Reduction
Source: Polymers (Basel). 2020 May 14;12(5):1120. doi: 10.3390/polym12051120 (PMC7284564; doi:10.3390/polym12051120)
Supplement: Supplementary file 1 [file polymers-12-01120-s001.pdf]

Supplementary

A Biomimetic Approach to Increasing Soft Actuator Performance by Friction Reduction

Nguyen Quang Khuyen [1], Rudolf Kiefer [1,*], Fred Elhi [2], Gholamreza Anbarjafari [3,4], Jose G. Martinez [5] and Tarmo Tamm [2]

1 Conducting polymers in composites and applications Research Group, Faculty of Applied Sciences, Ton Duc Thang University, 700000, Ho Chi Minh City, Vietnam; nguyenquangkhuyen@tdtu.edu.vn
2 Intelligent Materials and System Lab, Institute of Technology, University of Tartu, Nooruse 1, 50411 Tartu, Estonia; elhi.fred@gmail.com (F.E.); tarmo.tamm@ut.ee (T.T.)
3 iCV Research Lab, Institute of Technology, University of Tartu, 50411 Tartu, Estonia; shb@ut.ee
4 Faculty of Engineering, Hasan Kalyoncu University, 27100, Gaziantep, Turkey
5 Division of Sensor and Actuator Systems, Department of Physics, Chemistry and Biology (IFM), Linköping University, 581 83, Linköping, Sweden; jose.gabriel.martinez.gil@liu.se
* Correspondence: rudolf.kiefer@tdtu.edu.vn

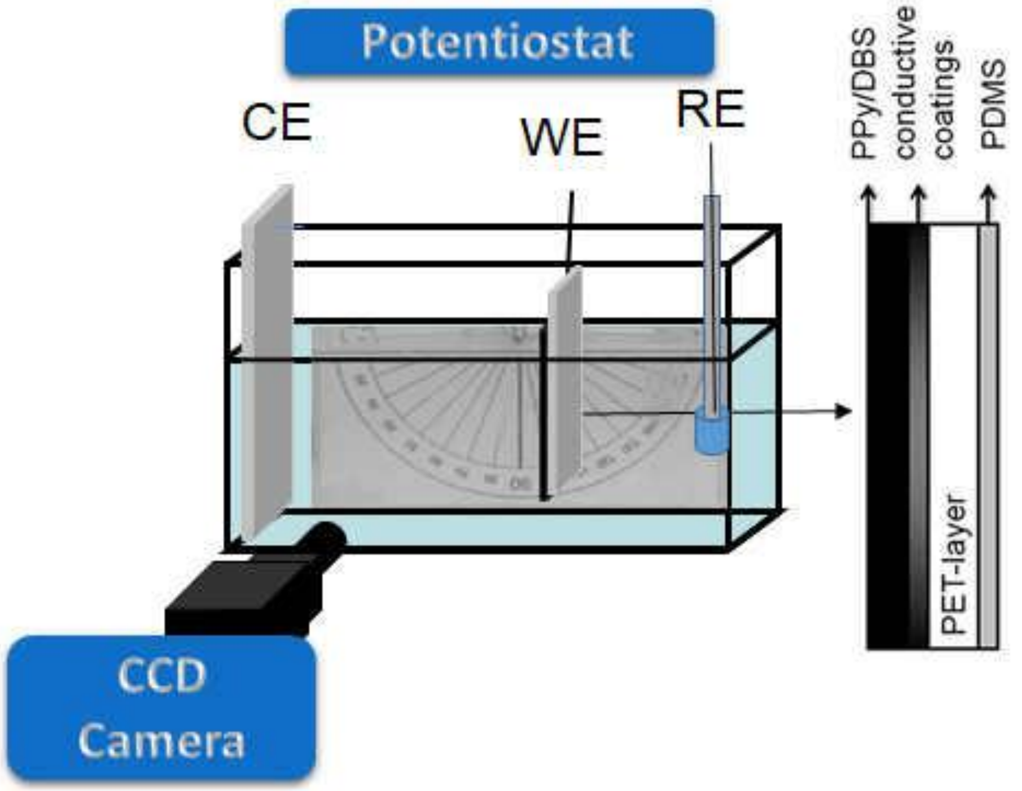

Figure S1. Set up of the bending beam measurements applying the bilayer samples as working electrode with counter electrode a platinum sheet and an Ag/AgCl (3M KCl) reference electrode in aqueous electrolyte.

A CCD camera (Sony Cyber-shot DSC-F717) connected to a PC installed frame grabber card was recording the bilayer movement. The scale in back shows the semicircle with s180 degrees scale, yielding the bending angle. Bending was recorded with a camera, the video then analyzed frame-by-frame with an in-house Matlab program. The principle assembling of the bilayer samples with and without the PDMS layer shown on right side.

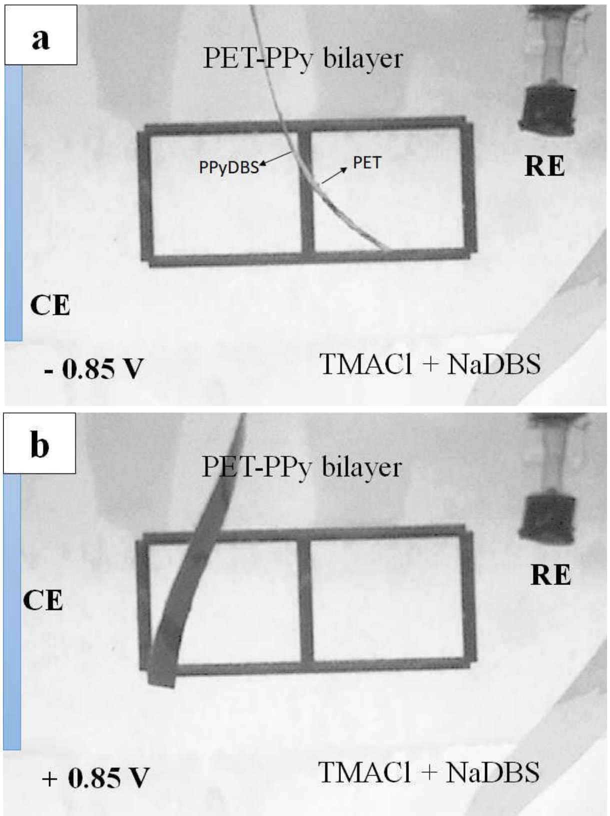

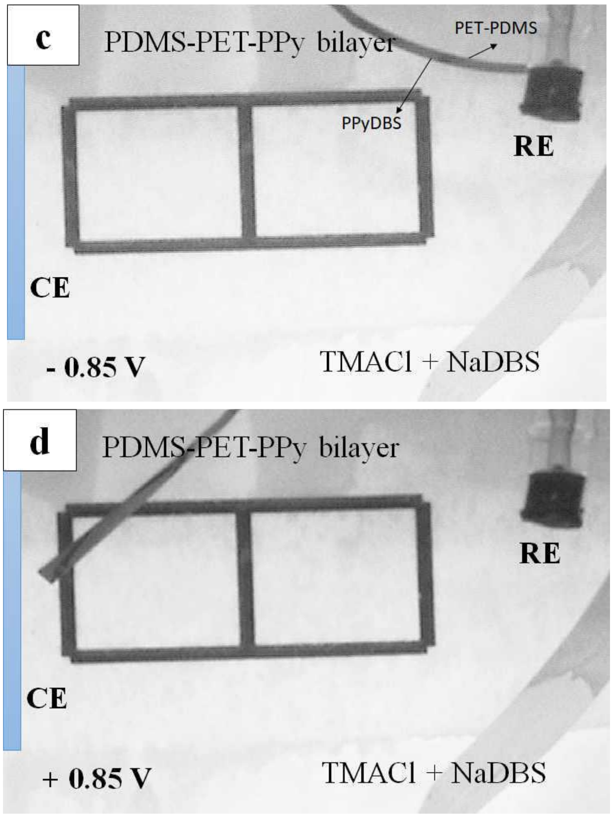

Figure S2. Videoframes of angular displacement under cyclic voltammetric measurements (scan rate 10 mV s^{-1}) in binary aqueous electrolyte (0.05 M NaDBS + 0.05 M TMACl) with Pt counter electrode (CE) and Ag/AgCl (3M KCl) reference electrode (RE) showing the images of a: PET-PPy bilayer at -0.85 V, b: PET-PPy-bilayer at +0.85 V, c: (PDMS)PET-PPy bilayer at -0.85 V and PDMS-PET-PPy bilayer at +0.85 V. The position of the PET side and PPy/DBS side is shown in (a) and for (PDMS)PET-PPy bilayer in (c).

The scale inside the images are 2 cubes with 1cm * 1cm dimension. The images can be read out by using frames of the recorded video over a graphical program. So far we have applied angle where no inner scale needed as the computer program make a 90 degree starting position and read out the change in angle during displacement.

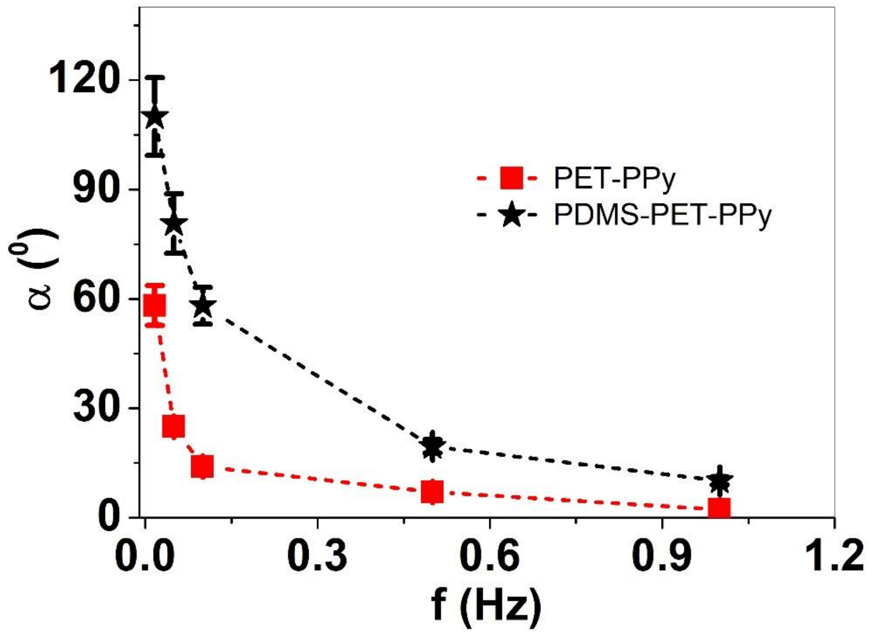

Figure S3. The angular displacement α of PET-PPy (■) and PDMS-PET-PPy (★) at square wave potential steps against the applied frequency f (0.017 Hz to 1 Hz). .
